# Editorial: Gaps, priorities, and advances in cardiovascular medicine in Africa

**DOI:** 10.3389/fcvm.2023.1260406

**Published:** 2023-08-09

**Authors:** Ruan Kruger, Augustine Odili, Lebo F. Gafane-Matemane

**Affiliations:** ^1^ Hypertension in Africa Research Team (HART), North-West University, Potchefstroom, South Africa; ^2^ MRC Research Unit for Hypertension and Cardiovascular Disease, North-West University, Potchefstroom, South Africa; ^3^ Department of Internal Medicine, Faculty of Clinical Sciences, College of Health Sciences, University of Abuja, Abuja, Nigeria

**Keywords:** Africa, cardiovascular health, cardiovascular disease, medicine, innovation, priorities, policy

**Editorial on the Research Topic** Gaps, priorities and advances in cardiovascular medicine in Africa

Africa has been a research hub for many scientific and clinical investigations from the global North but remains one of the regions with the greatest disproportions in health equity and resources (1). Among several communicable and non-communicable disease (NCD) challenges, cardiovascular disease (CVD) is a major population health and economic burden with increasing morbidity and mortality rates (2). On the African continent, CVD ranges from atherosclerotic disease, cardiomyopathy, congenital heart disease, hypertension, and rheumatic heart disease, to tuberculous pericarditis, among others (3, 4). Opportunities are available to change the CVD epidemiology trajectories since several gaps have been reported in the improvement of CVD management and awareness in Africa. However, these changes are only possible when priorities are identified and addressed by policymakers, functional health systems and engaging communities.

With the current escalating burden of CVD in Africa, this Research Topic aimed to collate available information and provide insights into the current gaps and priorities in cardiovascular medicine in African countries, calls to action for the current CVD challenges in Africa and research advances in the broader field of cardiovascular medicine in Africa. This Research Topic is therefore dedicated to highlighting research performed in Africa and highlighting the limitations and opportunities in the health sector of African countries.

The limited resources allocated to prevention and control of NCDs such as hypertension and type 2 diabetes mellitus in sub-Saharan Africa threatens to reverse the gains made in increasing life expectancy through the rapid scale-up of care for communicable diseases (5). In the review of the current state of CVD in Africa, Minja et al. present a comprehensive profile of the current cardiovascular pathologies in sub-Saharan Africa, with heart failure, atherosclerotic cardiovascular diseases, coronary artery disease, and stroke emerging as the main contributors to the CVD burden. As expected, the four major risk factors are high blood pressure, obesity, hyperglycemia, and tobacco use, with hypertension as the leading cause of stroke, ischemic, and hypertensive heart disease in all four sub-Saharan Africa regions (West, East, Central, and Southern). The identified essential gaps in this review are the generation of representative data on CVD prevalence and outcomes, limited healthcare workforce, poor access to CVD care, and low education and literacy levels at an individual level. Relevant priority areas include the generation of evidence on preventative and control strategies that will inform implementation into practice, capacity building in the healthcare sector as well as improved and equitable access to CVD care.

In line with these key priority areas, Chillo et al. discuss some of the opportunities that can be leveraged to improve CVD research in Tanzania, which is tasked with developing the East African Centre of Excellence in Cardiovascular Sciences (EACoECVS). The goal of the center is to contribute to the prevention and control of CVD and subsequently morbidity and mortality in Tanzania and the East African region. This is to be achieved by tackling the following priorities: generation of CVD burden data, prevention and health promotion research, research on early detection and management of CVD, establishing disease-specific registries along with harnessing the big data available from daily hospital records, and lastly research on the genetic aspects of CVD development and progression. The strategies target academia, as an essential environment to conduct research, develop expertise and contribute to quality research outputs. The presented strategies address challenges relating to the training of CVD researchers in SSA and the availability of infrastructure for training and conduction of research as well as research funding. The EACoECVS has already started work on mitigating these challenges for sustainable CVD research in Tanzania and the region.

The study by Agbo et al. highlighted an ongoing challenge in the accurate assessment of abdominal obesity in the Southern African landscape. For decades, researchers revealed the inappropriate use of body mass index (BMI) as a diagnostic tool to evaluate overweight and obesity status. While the use of BMI is still endorsed by the World Health Organization, the Nigerian team from the Okeahialam group at the Jos University Teaching Hospital innovatively proposed another tool to assess abdominal obesity more accurately in sub-Saharan Africa. The so-called abdominal height is measured using an abdominometer and yielded a larger area under the curve and stronger sensitivity (than BMI and waist/hip ratio) for the detection of abdominal obesity for both men and women, but specificity was poor.

In sub-Saharan Africa, hypertension and human immunodeficiency virus (HIV) are some of the highest ranked burdens of non-communicable and communicable diseases, respectively (6). With the effectiveness of antiretroviral therapy, the longevity of people living with HIV is prolonged, contributing to a higher risk of developing hypertension and advanced CVD. A review paper from Mutengo et al. focused on a key mechanism that may potentially play a role in contributing to salt-sensitive hypertension in patients with HIV, namely altered epithelial sodium channel (ENaC) activity. While ENaC is known for its role in blood pressure regulation through renin-angiotensin-aldosterone system activation, extrarenal factors such as oxidative stress can also enhance ENaC activity. Similarly, oxidative stress has a strong involvement in the development of hypertension and target organ damage by forming Isolevuglandins and activating pro-inﬂammatory cascades. Sex-specific differences in antiretroviral therapy and HIV are largely unexplored, but certain sex differences may contribute to hypertension. Sodium storage in women is mostly in muscle tissue, while men store sodium in the skin which can induce proinflammation. Women are also more prone to develop hypertension due to their higher susceptibility for salt sensitivity, diet induced dysbiosis and use of combined oral contraceptives. From the same group, Masenga et al. reported original work on sex differences in hypertension among people living with HIV after the initiation of antiretroviral therapy. They reported that BMI and the use of protease inhibitors are strongly associated with incident hypertension, especially in men.

In this research topic, a study protocol was published to assess cardiovascular risk profiles in African ancestry infants exposed to in-utero pre-eclampsia Nkeh-Chungag et al. With mounting evidence pointing toward the early life exposures of maternal risk factors and future premature onset of CVD in predisposed offspring, this is a timely study to be executed on African soil. This is especially of importance since non-African studies reported that children born to pre-eclamptic mothers are prone to develop obesity, hypertension, and diabetes in adult life—which remains to be established in the African context.

Not many papers were submitted to this Research Topic, highlighting the potential limitations in the African setting regarding resources and capacity to optimise efforts in addressing health equity. More work is encouraged in Africa by Africa with a strong focus to develop Africa-centric nomograms, thresholds, clinical practice guidelines and knowledge in cardiovascular medicine.
